# Supplementary material for: Entanglement of photonic modes from a continuously driven two-level system
Source: npj Quantum Inf. 2025 Apr 28;11(1):69. doi: 10.1038/s41534-025-00995-1 (PMC12037412; doi:10.1038/s41534-025-00995-1)
Supplement: Supplementary file 1 — Entanglement of photonic modes from a continuously driven two-level system—Supplementary Material [file 41534_2025_995_MOESM1_ESM.pdf]

Entanglement of photonic modes from a continuously driven two-level system — Supplementary Material

Jiaying Yang[1,2], Ingrid Strandberg[1], Alejandro Vivas-Viaña[3,4], Akshay Gaikwad[1], Claudia Castillo-Moreno[1], Anton Frisk Kockum[1], Muhammad Asad Ullah[2], Carlos Sánchez Muñoz[3,4], Axel Martin Eriksson[1], Simone Gasparinetti[1]

[1]*Department of Microtechnology and Nanoscience, Chalmers University of Technology, SE-412 96, Göteborg, Sweden*
[2]*Ericsson Research, Ericsson AB, SE-164 83, Stockholm, Sweden*
[3]*Departamento de Física Teórica de la Materia Condensada and Condensed Matter Physics Center (IFIMAC), Universidad Autónoma de Madrid, 28049 Madrid, Spain*
[4]*Institute of Fundamental Physics IFF-CSIC, Calle Serrano 113b, 28006 Madrid, Spain*

I. MEASUREMENT DETAILS

In this work, we utilize a $6.6 \times 6.6\,\mathrm{mm}^2$ superconducting device. The device is fabricated on a silicon substrate, and its RF lines and ground plane consist of aluminium layers deposited on top of the substrate. We wire-bond the device in a copper sample holder, which is then enclosed within a copper shield. To provide additional protection against magnetic interference, we place the bonded device inside another μ-metal shield (cryoperm). This shielding is installed in the mixing chamber of a dilution refrigerator to ensure that all experimental measurements are conducted at temperatures below 15 mK (Fig. S1).

We use the same device as in our previous work [1] in this experiment. However, only the emitter qubit (orange) and the waveguide (red) are used in this work. The anharmonicity of the emitter is $\alpha/2\pi = 220\,\mathrm{MHz}$. The unused coupler (not shown) on the device is coupled to the qubit, but has no impact due to its high frequency, 7.735 GHz, far away from the emitter. The waveguide capacitively coupled to the emitter connects to the reflection input and output lines on the other side, from where the emitted photon field is measured via a traveling-wave parametric amplifier (TWPA) [2] and a high-electron-mobility transistor (HEMT) amplifier in the output line. In our system, we measure the quantum efficiency η to be 0.043, where $\eta = \frac{1/2}{1/2+n_{\mathrm{added}}}$ and $n_{\mathrm{added}} = 11$ is the added noise photon number by the amplification chain. Aside from the coplanar waveguide, the emitter also capacitively couples to a charge line, which is grounded and not used in this work.

In the experiment, data are obtained using a pulsed setup. Microwave control pulses are sent to drive the emitter using arbitrary waveform generators (AWGs), and the data are read out with analogue-to-digital converters (ADCs) from the microwave transceiver platform Vivace [3], after up- and down-conversion by IQ mixers and local oscillators. Temporal mode matching and data acquisition begin when the qubit drive has lasted for $t_0 = 200\,\mathrm{ns}$—a duration much longer than the qubit's relaxation time T_1—ensuring the qubit is in a steady state.

II. THEORETICAL RESULTS ON ENTANGLEMENT GENERATION

In this section, we extend Results by simulating the logarithmic negativity and the two-photon correlation function at zero delay in the frequency domain. We study the effects of varying the driving strength of the microwave pulse (Ω), the frequency of the filters (Δ_k), and the duration of the measurement (T) [see Fig. S2(a-h)].

A. Entanglement in the frequency domain

Throughout this work we focused on the specific case where the output modes correspond to opposite sidebands. However, by extending the range of frequencies in the filtering process, we unveil a rich structure in the generation of entanglement, as depicted in Fig. S2(a, b). In the frequency map [upper-left triangle in Fig. S2(b)], we observe two main regions of non-zero logarithmic negativity: the diagonal ($\Delta_1 = \Delta_2$) and the antidiagonal ($\Delta_2 = -\Delta_1$). However, only the later is relevant to this work, as discussed below.

The diagonal section entails a breakdown of the orthogonality assumption between modes, which we consider a basic requirement for discussing entanglement between two different systems. More generally, this assumption will be compromised in sections where $|\Delta_1 - \Delta_2| < \Delta\omega$ or $\Omega < \Gamma$—where $\Delta\omega$ is the frequency resolution inherited by the filter—since the reported values of entanglement cannot be understood as actual measures of entanglement. In these cases, spectral resolution is lost and the collected modes become indistinguishable in frequency. Nevertheless, this information reveals the amount of entanglement achievable if the whole a_{out} is physically split into two orthogonal

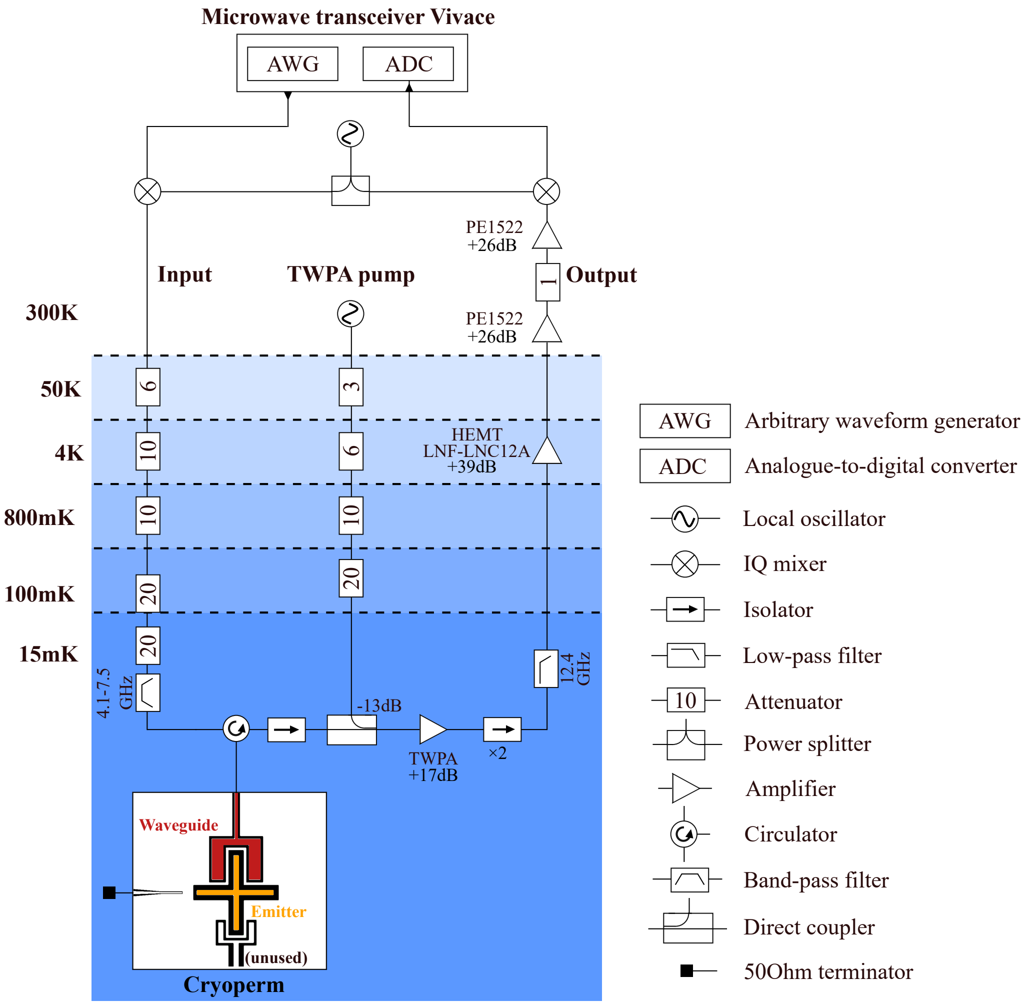

FIG. S1. Wiring diagram of the system, showing how the superconducting chip is connected to the input line and output line of the experimental setup. The drive signal to the emitter is applied through the input line, reaches the emitter through the coplanar waveguide, and the output signal is measured through the output line of the system. Note that the output from the AWG and the input to the ADC has two ports, including the in-phase component (I) and the out-of-phase component (Q), but only one port is shown in the figure for simplicity. The microwave transceiver board Vivace [3] provides both AWG and ADC channels.

modes (e.g., by a beam splitter) prior to filtering, in which case entanglement between any two pair of temporal modes extracted from each of the split outputs would be well-defined. As discussed in Methods A, this requires a slightly different master equation accounting for the vacuum contributions introduced by splitting the signal, with results strongly correlated to those presented here (not shown).

Therefore, regimes in which $\Omega > \Gamma$ and $|\Delta_1 - \Delta_2| > \Delta\omega$ are the most interesting since it provides a well-resolved emission in different spectral lines, resulting in well-defined entanglement.

Additionally, in the lower panel in Fig. S2(b), we observe the photon-photon correlations of the filtered emission by computing the zero-delay two-photon cross-correlation

$$g^{(2)}_{1,2} = \frac{\langle \hat{a}_1^\dagger \hat{a}_2^\dagger \hat{a}_1 \hat{a}_2 \rangle}{\langle \hat{a}_1^\dagger \hat{a}_1 \rangle \langle \hat{a}_2^\dagger \hat{a}_2 \rangle}. \tag{S1}$$

As discussed in Ref. [4], this map exhibits the typical frequency structure of a coherently driven TLS, where the antidiagonal features a complex structure of correlations due to the multi-photon processes occurring at these frequencies. In fact, in Fig. S2(a), we observe a clear correspondence between entanglement and bunching around the frequency resonance, specifically at $(\Delta_1, \Delta_2) = (\Delta_-, \Delta_+)$.

Throughout this discussion, we have assumed that the two-mode temporal profiles, $v_1(t)$ and $v_2(t)$, are perfectly overlapping in time, while their frequencies remain independently tunable. Here, we investigate how relaxing the

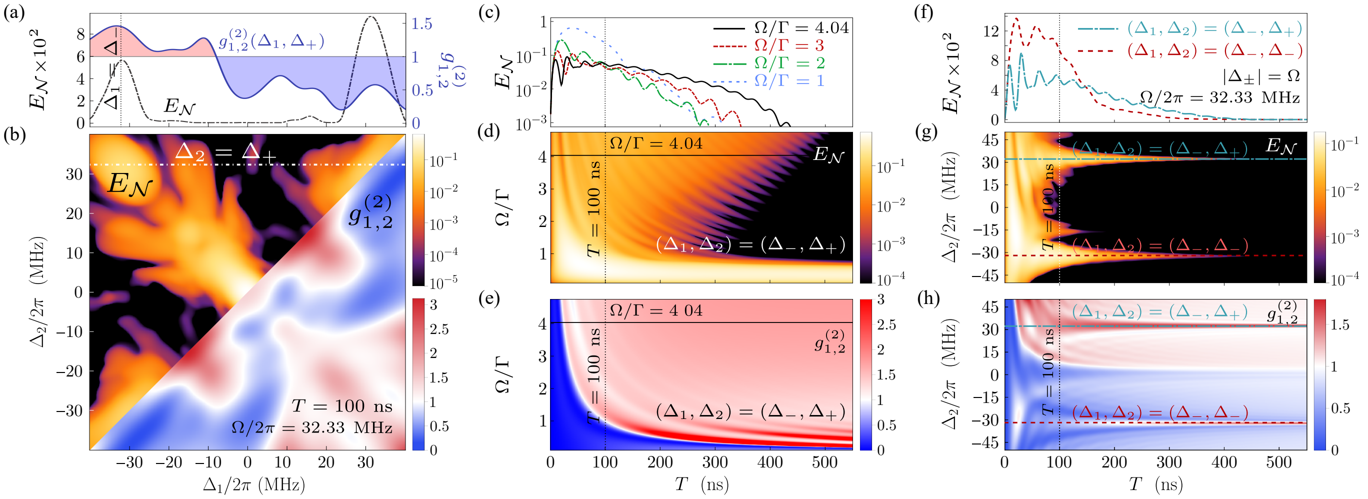

FIG. S2. Simulated logarithmic negativity (in logarithmic scale), $E_{\mathcal{N}}$, and two-photon correlation function at zero delay, $g^{(2)}_{1,2}$. (a) $E_{\mathcal{N}}$ (black dotted-dashed line) and $g^{(2)}_{1,2}(\Delta_1, \Delta_+)$ (blue solid line) in terms of the filter detuning Δ_1 [$\Delta_2 = \Delta_+$, white dotted-dashed line in (b)]. The vertical dotted line denotes the resonant frequency, $\Delta_1 = \Delta_-$. (b) $E_{\mathcal{N}}$ (upper-left triangle) and $g^{(2)}_{1,2}$ (lower-right triangle) in the frequency domain. Both figures are symmetric with respect the diagonal. (c) $E_{\mathcal{N}}$ at opposite sidebands [$(\Delta_1, \Delta_2) = (\Delta_-, \Delta_+)$, equivalently in (d,e)] in terms of the measurement time T for several pulse intensities $\Omega = \{1, 2, 3, 4.04\}\Gamma$. The latter is actually used in the experiment. (d,e) $E_{\mathcal{N}}$ and $g^{(2)}_{1,2}$, respectively, in terms of Rabi frequency of the drive Ω and the measurement time T. In (e), for practical reasons, the data is upper-bounded up to 3. The actual maximum value, ≈ 36, only occurs at low pumping and long filtering times (bottom-right red areas). (f) $E_{\mathcal{N}}$ in terms the measurement time T for the two sideband resonances, $(\Delta_1, \Delta_2) = (\Delta_-, \Delta_\pm)$, in blue dot-dashed and red dashed lines, respectively [equivalently in (g,h)]. (g,h) $E_{\mathcal{N}}$ and $g^{(2)}_{1,2}(\Delta_-, \Delta)$, respectively, in terms of the filter detuning Δ_2 and the measurement time T. The vertical dot-line and horizontal solid line in (c–h) correspond to the experimental values used in the experiment: $(\Omega, T) = (32.32\ \text{MHz}, 100\ \text{ns})$. Parameters: $\Gamma/2\pi = 8$ MHz, $\Omega/2\pi = 32.33$ MHz, $\Delta = 0$, $t_0 = 200$ ns, $T = 100$ ns.

requirement of perfect temporal overlap influences the entanglement. Specifically, we analyze a scenario in which the boxcar-shaped temporal filters applied to the modes are temporally offset from each other, introducing a controllable time delay T_{Delay} between them. We perform simulations where the starting time of the filter for the second temporal mode (TM2) is adjusted to $t_0 + T_{\text{Delay}}$, with T_{Delay} varied within the range $[-0.125, 0.125]\,\mu$s (Fig. S3). The initial starting time of the other mode (TM1) is fixed at t_0. Additionally, the duration of the templates is systematically varied to examine its influence. The logarithmic negativity $E_{\mathcal{N}}$, measured at the maximum-entanglement point $(\Delta_1, \Delta_2) = (\Delta_-, \Delta_+)$, exhibits a symmetric decaying pattern centered around $T_{\text{Delay}} = 0$, implying that for any duration, entanglement is maximized by choosing the two modes to perfectly overlap in time.

B. Effects of the filtering time

The dependence on T in all the results in Fig. S2(c-h) exhibits similar behaviour. The boxcar filter imposes an approximate bandwidth of $\Delta\omega \sim 2\pi/T$—corresponding to the width of the central peak of the sinc function—such that in the limit of infinite frequency precision, $T \to \infty$, the filtered modes lose their time definition, causing entanglement to vanish. Conversely, very short measurement times, $T \to 0$, correspond to broadband detection (colour-blind) [see Fig. 2(b) in Results], resulting in a non-zero logarithmic negativity that does not reflect an actual measure of entanglement, as we previously discussed. Additionally, in this limit, we recover the expected antibunching of a two-level system. We also note that higher values of Ω extend the lifetime of the entanglement [see Fig. S2(c, d))].

In the limit of long T (i.e., very narrow frequency filtering), we observe that the photon statistics is not completely uncorrelated, $\lim_{T\to\infty} g^{(2)}_{1,2}(\Delta_-, \Delta_\pm) \neq 1$, but bunched ($\lesssim 2$) [see Fig. S2(e, h)]. This feature was already reported in Refs. [4–7], where it is shown that correlations between opposite sidebands feature bunching when a perfect laser is used to drive the TLS. To recover the expected limit of uncorrelated photons when $\Delta_1 \neq \Delta_2$, a more realistic model of a laser must be considered, such as an one-atom laser [8]. In Fig. S2(f, g), we see that when one of the filters is fixed on a Mollow sideband, e.g., $\Delta_1 = \Delta_-$, the collected emission by the other filter features two main resonances that contribute to the generation of entanglement. These resonances correspond to (i) opposite sidebands, $(\Delta_1, \Delta_2) = (\Delta_-, \Delta_+)$ (in blue), and (ii) identical sidebands, $(\Delta_1, \Delta_2) = (\Delta_-, \Delta_-)$ (in red). However, as previously

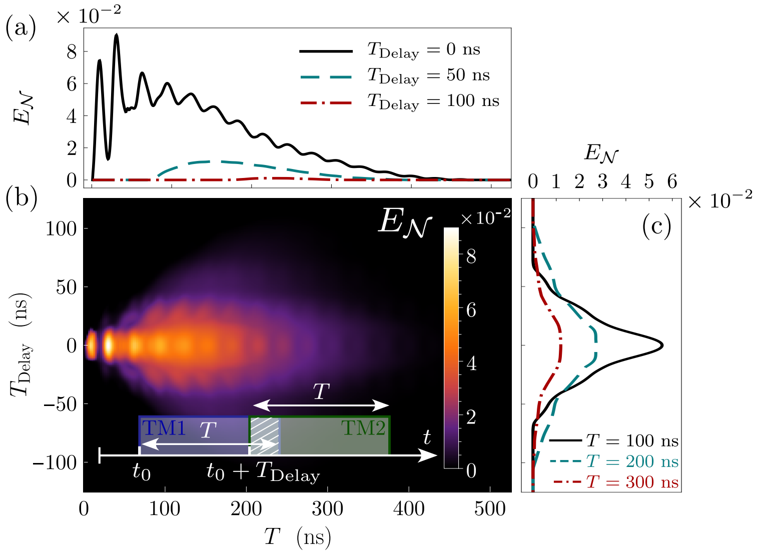

FIG. S3. Simulated logarithmic negativity $E_{\mathcal{N}}$, evaluated as a function of the time delay between two temporal boxcar filters and their durations. (a) Map of $E_{\mathcal{N}}$ as a function of both starting-time delay $T_{\rm Delay}$ and duration T. The inset illustrates the time sequence of the applied temporal filters: the first filter, TM1, starts at a fixed time t_0, while the starting time of the second filter, TM2, is varied. (b) Dependence of $E_{\mathcal{N}}$ on T for selected values of $T_{\rm Delay}$. (c) Dependence of $E_{\mathcal{N}}$ on $T_{\rm Delay}$ for selected values of T. Entanglement decreases as the delay is increased.

discussed, the nature of these entangled states differs since only the first case involves entanglement between two distinct, well-resolved light modes.

C. Entanglement in the time domain

Besides orthogonal frequency modes with a boxcar temporal filter, there are other options for the mode shapes. A common temporal mode basis is the *Hermite–Gauss* modes. They have certain overlap in both time and frequency, but are field-orthogonal [9, 10]. The mode shape is defined as

$$f_i(t) = H_n\left(\frac{t}{w}\right)\exp\left(-\frac{t^2}{2w^2}\right), \tag{S2}$$

where H_n is the nth Hermite polynomial, and w determines the temporal width of the mode. Index $i = 1, 2$ indicates our previous mode filter numbering. We simulate logarithmic negativity for Hermite–Gauss modes with $n = 0$ and $n = 1$ using $w = 0.5$ in Fig. S4. The maximum logarithmic negativity ($E_{\mathcal{N}} = 0.155$) is more than twice as large as that of the boxcar modes ($E_{\mathcal{N}} = 0.062$). This improvement in logarithmic negativity indicates potential advantages of exploring alternative mode shapes beyond boxcar modes.

III. TWPA GAIN COMPENSATION

To calibrate the gain of the TWPA, we conduct a single-tone spectroscopy analysis of the qubit through the waveguide. This is achieved under the condition that the qubit is saturated by utilizing high power in the microwave input pulse. The spectroscopy is performed twice across the bandwidth of $[-40, 40]$ MHz around the qubit's resonance frequency, both with the TWPA powered and unpowered. We obtain the on-off gain of the TWPA by calculating the ratio between the spectroscopy measured in the two cases [Fig. S5(a)].

Due to the observed variation in TWPA gain across the specified bandwidth, we apply compensation to both temporal-matched one-mode and two-mode data of the propagating modes. One-mode data (see Fig. 2 in Results) is compensated by dividing the data with the gain trace in Fig. S5(a). For two-mode data (see Fig. 3 in Results) which are two-dimensional matrices for both modes $\hat{a}_1$ and $\hat{a}_2$, we map the gain trace into gain matrices according to Fig. S5(b), allowing us to compensate $\hat{a}_1$ and $\hat{a}_2$ by dividing the corresponding gain matrices.

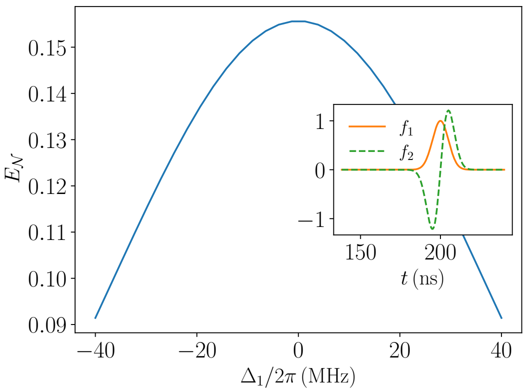

FIG. S4. Simulated logarithmic negativity for the first two Hermite–Gauss modes $n = 0, 1$ in Eq. (S2) with $w = 0.5$. The detuning Δ_1 of the first mode is varied while $\Delta_2 = 0$ is fixed. The entanglement is strongest when there is no detuning. Inset: The Hermite–Gauss modes in the time domain centered at t_0.

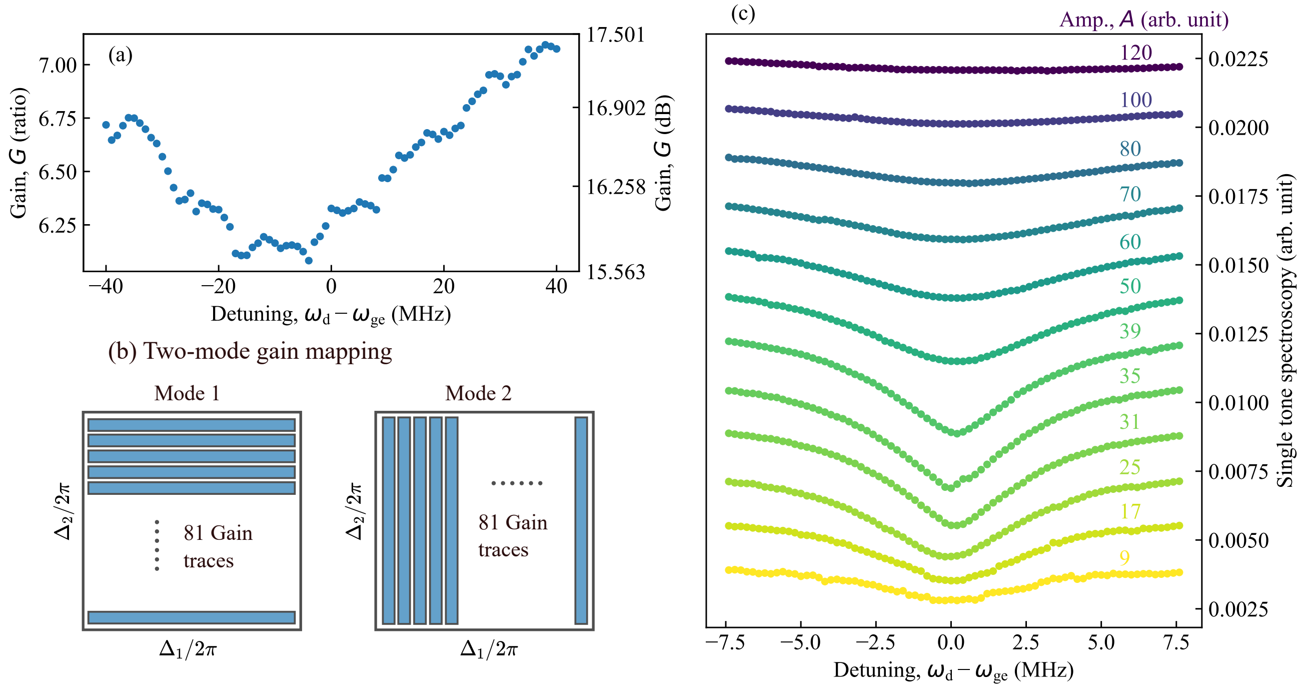

FIG. S5. TWPA gain and AWG amplitude calibration for the system. (a) The on-off gain of the TWPA as a function of the detuning between the drive frequency ω_d and the qubit frequency ω_ge, presented in ratio and with unit dB. (b) Schematic diagram showing how we map the gain trace in (a) into gain matrices, for two-mode data compensation. The same time trace is repeated horizontally (vertically) for mode 1 (2) data $\hat{a}_1$ ($\hat{a}_2$). Each gain matrix contains 81 gain traces, corresponding to the number of points along the frequency detuning axes. (c) The single-tone spectroscopy of the qubit measured with ADC, when applying microwave pulses with different driving amplitude from AWG. Each trace is normalized with the corresponding driving amplitude and is offset by 0.0017 compared to the trace below it.

IV. CONVERSION BETWEEN AWG AMPLITUDE TO RABI RATE

We obtain the conversion between the amplitude of the AWG output pulse and the drive Rabi frequency Ω by operating power calibration of the qubit [Fig. S5(c)]. We drive the qubit with pulses from the AWG with different amplitudes, and measure the single-tone spectroscopy of the qubit from the reflection setup connected to the waveguide. At the critical power of the qubit, where the qubit-driving power maximizes the coherence between the ground and excited states, the strength of the input pulse is $\Omega = \Gamma/\sqrt{2}$ [11]. From the measurement, we observe that when the driving amplitude is $A_\mathrm{critic} = 35$ (arb. unit), the qubit spectroscopy has the largest dip, corresponding to the case

with the critical power. Due to the proportional relationship between the drive rate Ω and the driving amplitude A_{in}, any strength can therefore be converted from the input power P_{in} of the driving pulse by following

$$\Omega = \frac{A_{\text{in}}}{A_{\text{critic}}} \frac{\Gamma}{\sqrt{2}} . \tag{S3}$$

[1] J. Yang, A. M. Eriksson, M. A. Aamir, I. Strandberg, C. Castillo-Moreno, D. P. Lozano, P. Persson, and S. Gasparinetti, Deterministic generation of shaped single microwave photons using a parametrically driven coupler, Physical Review Applied **20**, 054018 (2023).
[2] C. Macklin, K. O'Brien, D. Hover, M. E. Schwartz, V. Bolkhovsky, X. Zhang, W. D. Oliver, and I. Siddiqi, A near–quantum-limited Josephson traveling-wave parametric amplifier, Science **350**, 307 (2015).
[3] Intermodulation Product, *Vivace microwave platform*.
[4] A. Gonzalez-Tudela, F. P. Laussy, C. Tejedor, M. J. Hartmann, and E. del Valle, Two-photon spectra of quantum emitters, New Journal of Physics **15**, 033036 (2013).
[5] A. Aspect, G. Roger, S. Reynaud, J. Dalibard, and C. Cohen-Tannoudji, Time Correlations between the Two Sidebands of the Resonance Fluorescence Triplet, Physical Review Letters **45**, 617 (1980).
[6] J. Dalibard and S. Reynaud, Correlation signals in resonance fluorescence : Interpretation via photon scattering amplitudes, Journal de Physique **44**, 1337 (1983).
[7] G. Bel and F. L. H. Brown, Theory for Wavelength-Resolved Photon Emission Statistics in Single-Molecule Fluorescence Spectroscopy, Physical Review Letters **102**, 018303 (2009).
[8] Y. Mu and C. M. Savage, One-atom lasers, Physical Review A **46**, 5944 (1992).
[9] B. Brecht, D. V. Reddy, C. Silberhorn, and M. G. Raymer, Photon Temporal Modes: A Complete Framework for Quantum Information Science, Physical Review X **5**, 041017 (2015).
[10] M. G. Raymer and I. A. Walmsley, Temporal modes in quantum optics: Then and now, Physica Scripta **95**, 064002 (2020).
[11] M. Scigliuzzo, A. Bengtsson, J.-C. Besse, A. Wallraff, P. Delsing, and S. Gasparinetti, Primary thermometry of propagating microwaves in the quantum regime, Physical Review X **10**, 041054 (2020).
